# Factors associated with stunting and wasting in children under 2 years in Bangladesh

**DOI:** 10.1016/j.heliyon.2020.e04849

**Published:** 2020-09-14

**Authors:** Tuhinur Rahman Chowdhury, Sayan Chakrabarty, Muntaha Rakib, Sabiha Afrin, Sue Saltmarsh, Stephen Winn

**Affiliations:** a Department of Economics, Shahjalal University of Science & Technology, Sylhet Kumargaon, Sylhet 3114, Bangladesh; b University of Southern Queensland, Springfield, QLD 4300, Australia; c Department of Early Childhood Education, The Education University of Hong Kong, Tai Po Campus, New Territories, Hong Kong; d School of Education, Edith Cowan University, Australia

**Keywords:** Public health, Determinants, Developing country, Growth failure., Chronic malnutrition, Child undernutrition, Socioeconomic risk factors, Acute malnutrition

## Abstract

Child undernutrition has been a major concern for Bangladesh as it is amongst the highest stunting and wasting prevalent countries in the world. The objective of our study was to explore the socioeconomic determinants of stunting and wasting in children under two years. This study explored nationally representative sample of 7,230 children ranging in age from 0 to <24 months using two separate binary logistic regression models to determine the risk factors associated with child stunting and wasting. Our study estimated approximately 33 percent children to be stunted and 11 percent to be wasted. Our analysis found that, 12 to <24 months old children's height-for-age-z-score and weight-for-height-z-score deteriorated in comparison to those of below 6 months. Female children had significantly lower odds of stunting and wasting compared with male children. Study revealed that children from wealthier families were at lower risk of being stunted and wasted compared to children from poorer households. Parental education was determined as a significant predictor of stunting. Children who lived in Sylhet division were 1.26 times more likely to be stunted than the children of Dhaka division [OR = 1.26; 95% CI: 1.02–1.55].

Our study revealed age, gender, geographic distribution, and household's position in wealth index as common determinants of child stunting and wasting in Bangladesh. While parental education was significant predictor for child stunting, type of toilet facility was found as statistically significant determinant of child wasting in children of less than two years age.

## 1 Introduction

Throughout the world, children are likely to be healthier and have linear growth when they are properly nurtured and nourished with adequate and nutritional dietary intake. In 2016, 5.6 million children under the age of 5 years died, with 45% of these deaths attributed to undernutrition (WHO, 2016). Globally, more than 200 million school-age children are stunted and it is predicted that nearly 1 billion children will grow up with impaired physical and cognitive health by 2020 unless this problem is addressed (The World Bank, 2012). Stunting has both short and long term and direct and indirect effects on individual children, contributing to low birth weight, hindering cognitive development and school achievement, and limiting their life prospects into adulthood. It also has significant national implications, including child mortality rates and economic productivity (Victoria et al., 2008; Dewey and Begum, 2011). Stunting is an underlying cause of children's death and disease burden which accounts for about 3.1 million or 45% of global child death per annum apart from diseases and disability (Black et al., 2013; United Nations Children's Fund (UNICEF), 2016; Black et al., 2008). Stunting is claimed to place heavy burden on socio-economic and healthcare system (Tydeman-Edwards et al., 2018). By comparison, approximately 52 million children in the world under-five years of age were wasted in 2011 which is 11% improvement from that of 1990 (United Nations Children's Fund (UNICEF), 2012). Wasting accounts for an estimated 13% of child death globally (McCuskee et al., 2018).

However, reduced height for age or stunting is more predominant than underweight (low weight for age, 20%) or wasting (low weight for height, 10%) in developing countries (Dewey and Begum, 2011). The reason behind this is probably food intake, given that dietary quality is directly associated with height in growing age. There is also increasing evidence that interrupted early linear growth implies impaired adult growth, especially body size and intellectual functioning (Martorell et al., 2010). Infants and children under five are particularly susceptible to undernutrition in developing countries despite an overall decrease of stunting rate in the recent decades. For example, from 1970 to 2010 stunting reduced by 20% in all developing countries even though the prevalence varies between countries (Smith and Haddad, 2015). Still, stunting is in the highest frequency (40%) in South Asia and Sub-Saharan Africa (Smith and Haddad, 2015). Similarly, 70% of the world's wasted children live mostly in South-Central Asia.

Like other low and middle-income countries, child malnutrition remains a major public-health problem in Bangladesh and is a prevalent cause of death of children under five. In Bangladesh, the mortality rate of children under five decreased from 88 to 38 per 1,000 live births from 2004 to 2015 (BDHS, 2014). Despite the significant long-term decline in infant mortality rate, it is still very high (United Nations Children's Fund (UNICEF), 2016). The period from 0 – 24 months is of great importance for ensuring children's optimum growth and overall health. Nutritional deficiencies during this early period of life hamper both optimal physical growth and cognitive development, the implications of which continue into adulthood. Although, many studies have been conducted in Bangladesh on determining risk factors associated with stunting and wasting among children less than five years (Ahmed et al., 2016; Choudhury et al., 2000; Semba et al., 2008), studies for children below two years have been very limited (Choudhury et al., 2016, [Bibr bib13][Bibr bib12]; [Bibr bib2][Bibr bib13]). We, therefore, conducted this study to address this research gap.

The main objectives of our study were to identify the risk factors associated with the two categories of under nutrition-stunting and wasting among Bangladeshi children aged less than 24 months, and to identify policy, program and investment priorities in the context of Bangladesh.

## 2 Methods

Data used for our study were taken from Bangladesh Multiple Indicator Cluster Survey (MICS 2012–13), a nationally representative survey conducted by Bangladesh Bureau of Statistics with technical and financial support from United Nations Children's Fund (UNICEF) in Bangladesh. This survey provides the most recent data on a wide range of indicators on maternal and child health, child development and household characteristics on seven divisions and 64 districts. The more detail description of study setting, sampling techniques and data collection procedures can be found on ‘Bangladesh Multiple Indicator Cluster Survey 2012–13, ProgotirPathey: Final Report” that can be accessed on https://www.unicef.org/bangladesh/MICS_Final_21062015_Low.pdf.

In brief, a total of 55,200 households were selected as the survey sample using a two-stage stratified cluster sampling approach. There were 64 districts at the time of data collection and districts were defined as the sampling strata. A specific number of census enumeration areas were selected within each stratum using probability proportional to size (PPS), which was defined as primary sampling units (PSUs). Listing of households was updated within each enumeration area and a total of 20 households were drawn from each enumeration area using a systematic random sampling technique. Structured questionnaires were used for collecting data. There were four questionnaire sets:

(1)Household questionnaire set which was used to collect data on household members, household characteristics, water and sanitation, and the consumption of iodized salt; (2) Under-five questionnaire was used for collecting information on child age, anthropometry, early childhood development and breastfeeding; (3) Women's questionnaire (aged 15–49) collected information on women's marriage, child mortality, maternal and newborn health, postnatal health care, etc.; (4) A water testing questionnaire was used to determine the presence of arsenic in household drinking water. This set of questionnaires was administered to a subset of sample households. Prior to data collection, data collectors were provided with specific training that included lectures on data collection methods, including techniques for conducting interviews with respondents. Data were collected between December, 2012 and April, 2013. Information on 20,402 children below five years old was collected. As our interest in our study was on children below 24 months, we used only data pertaining to children aged less than 24 months for our analysis. Children with no information on either height-for-age z-scores or on weight-for-height z-scores were excluded from our study. Data pertaining to a total of 7,230 children below 24 months were subject to our analysis.

We developed two separate models for our study. The first of these models was to estimate the factors associated with the risk of stunting, and the second was to estimate factors associated with the risk of child wasting among the Bangladeshi children aged below 24 months. In defining stunting and wasting, we followed the WHO growth standard, with children whose height-for-age z-scores were below -2 being defined as stunted, and children whose weight-for-height z-scores were below -2 being defined as wasted (Saaka and Galaa, 2016). Our response variable in the first model was dichotomous by nature (stunted/not stunted). It is widely recognized that binary logistic regression model is more effective and accurate while analyzing binary data (Wilson and Lorenz, 2015). Logistic regression models are used to describe the relationship between a binary or categorical response variable and a set of predictor variables (Wilson and Lorenz, 2015). As our response variable was binary (stunted versus not stunted) and we wanted to describe the relationship between stunting and socioeconomic factors, we selected binary logistic regression model for our analysis. Similar to the first model, response variable for second model was dichotomous by nature (wasted/not wasted). We, therefore, also applied binary logistic regression model for our analysis in the 2^nd^ model. Predictor variables included in the models were: number of household members, children's age, gender, area, division, education of mother, education of father, type of toilet facility, salt iodization test outcome and the number of under-five children in household. We considered published articles and potential factors that might have impact on the risk of developing stunting and wasting while selecting predictor variables in our model. Although, ‘stunting’ and ‘wasting’ were two different indicators of malnutrition and they indicated two distinct physical impaired growth, we used the same variables for both model so that we could compare if there were any common significant factors that could have impact on the risk of developing stunting and wasting, and, thereby bringing recommendations to minimize that impact. We used STATA version 13 for our data analysis. A table with descriptive statistics was created. Statistical significance was set at p < 0.05 and the results from logistic regression were reported with odds ratio and 95% confidence interval. We applied Hosmer and Lemeshow's goodness of fit test to evaluate our model. We determined p value for first and second model to be 0.56 and 0.31 respectively. These p values indicate that our model fit the data. Multicolliniarity was tested with variance inflation factor (VIF). The mean VIF was estimated as 2.19 which was well below the maximum value of VIF at 10.

## 3 Results

### 3.1 Descriptive statistics

A total sample of 7,230 children below 24 months was used for analysis, of which the proportion of male children (50.7%) was almost the same as the proportion of female children (49.3%). A substantially larger percentage of study children lived in rural areas (84.1%) while only 16% lived in urban areas. Over half the study children lived in households which had pit latrines for defecation (62.0%). In salt iodization tests, only 52.8% households were found to have salt which was adequately iodized (15 ppm or more). The distribution of household socioeconomic status, measured by wealth index quintile, showed that the highest proportion of study participants (28.6%) lived in the poorest households and the lowest proportion of study participants (14.6%) lived in the richest households. Approximately half of the study population was aged 12 to <24 months (50.6%) and the proportion of children aged 0 to <6 months and 6 to <12 months was almost the same, 24.3% and 25.1% respectively. The highest proportion of children lived in Dhaka division (25.6%) and the lowest proportion of children lived in Sylhet division (9.2%). Only a small proportion of mothers (12.2%) had completed secondary or higher level education, and about one-fifth of mothers had no formal education (20.8%). The distribution of parental education in Table 1 suggests that the highest proportion of fathers (32.2%) had never attended school, and only 15.9% fathers had completed secondary or higher level education. 61.9% children lived in households with another household member 4 to <7 years, and only 12.3% children lived in households with another household member less than 4 years. The study estimated 33.4% children as stunted and 11.3% children as wasted.

**Table 1:** Socio-economic and background characteristics of the study population.

Characteristics	Total Sample
	(n = 7,230)
	Proportion (%)
**Household member**	
<4	12.3
4 to <7	61.9
7 or more	25.6

**Age (Months)**	
0 to <6	24.3
6 to <12	25.1
12 to <24	50.6

**Gender**	
Male	50.7
Female	49.3

**Area**	
Urban	16.0
Rural	84.1

**Division**	
Dhaka	25.6
Barisal	9.7
Chittagong	20.1
Khulna	13.8
Rajshahi	9.4
Rangpur	12.7
Sylhet	9.2

**Education of mother**	

Secondary complete or higher	12.2
Secondary incomplete	37.4
Primary complete	15.8
Primary incomplete	13.8
None	20.9

**Education of father**	
Secondary complete or higher	15.9
Secondary incomplete	21.4
Primary complete	14.7
Primary incomplete	15.9
None	32.2

**Wealth index quintile**	

Richest	14.6
Fourth	16.3
Middle	18.9
Second	21.7
Poorest	28.6

**Type of toilet facility**	
Flush latrine	24.6
Pit latrine	62.0
Hanging latrine	8.0
No facilities (field, bush)	5.4

**Salt iodization test outcome**	
Not iodized 0 PPM	26.9
More than 0 PPM and less than 15 PPM	20.3
15 PPM or more	52.8

**Stunting Condition**	

Stunted	33.4

**Wasting Condition**	

Wasted	11.3

### 3.2 Determinants of stunting

The prevalence of stunting was found to be highest (42.4%) among children aged 12 to <24 months [Table 2]. The proportion of male children who were stunted (35.7%) was higher than the proportion of female children who were stunted (31%). Sylhet had the highest proportion of stunted children (40.1%) and the lowest proportion of stunted children was in Khulna division (26.1%). Only 20.2% children who were found to be stunted lived in the richest households, while the prevalence rate climbed to 43.6% among those living in the poorest households. Households using flush latrines had the lowest proportion of stunted children (26.5%). As expected, the prevalence of stunting was lowest (17.9%) among children of mothers who had completed secondary education, and the prevalence rate was highest among children of mothers who had no formal education (43.3%). Similar results were found for paternal education, with only 20.9% children of fathers who had completed secondary education being stunted.

**Table 2:** Risk factors for stunting (HAZ scores < -2SD) among children aged less than twenty four months.

Factors		Stunted (%)	N	OR	95% CI	P value
Household member	<4	35.0	886	ref.		
	4 to <7	33.1	4,475	1.00	(0.84–1.18)	0.956
	7 or more	33.4	1,869	1.19	(0.97–1.45)	0.094
Age (Months)	0 to <6	23.2	1,758	ref.		
	6 to <12	25.2	1,813	1.13	(0.96–1.33)	0.151
	12 to <24	42.4	3,659	2.54	(2.21–2.92)	**<**0.001
Gender	Male	35.7	3,665	ref.		
	Female	31.0	3,565	0.78	(0.70–0.87)	**<**0.001
Area	Urban	30.2	1,153	ref.		
	Rural	34.0	6,077	0.88	(0.75–1.03)	0.115
Division	Dhaka	33.7	1,850	ref.		
	Barisal	30.4	677	0.77	(0.62–0.95)	0.014
	Chittagong	36.3	1,451	1.06	(0.90–1.25)	0.489
	Khulna	26.1	995	0.69	(0.57–0.84)	**<**0.001
	Rajshahi	30.0	677	0.78	0.64–0.96)	0.021
	Rangpur	36.0	916	1.03	(0.85–1.24)	0.795
	Sylhet	40.1	664	1.26	(1.02–1.55)	0.032
Education of mother	Secondary complete or higher	17.9	881	ref.		
	Secondary incomplete	29.6	2,705	1.47	(1.16–1.86)	0.002
	Primary complete	35.8	1,141	1.68	(1.28–2.20)	**<**0.001
	Primary incomplete	39.8	996	1.84	(1.39–2.43)	**<**0.001
	None	43.3	1,507	1.96	(1.49–2.59)	**<**0.001
Education of father	Secondary complete or higher	20.9	1,057	ref.		
	Secondary incomplete	30.7	1,426	1.29	(1.04–1.60)	0.019
	Primary complete	36.4	978	1.39	(1.10–1.75)	0.006
	Primary incomplete	37.4	1,060	1.31	(1.03–1.66)	0.027
	None	39.8	2,145	1.24	(0.98–1.55)	0.069
Wealth index quintile	Richest	20.2	1,056	ref.		
	Fourth	27.6	1,175	1.32	(1.04–1.67)	0.023
	Middle	30.1	1,364	1.28	(1.00–1.64)	0.055
	Second	36.0	1,571	1.52	(1.18–1.97)	0.001
	Poorest	43.6	2,064	1.96	(1.50–2.56)	**<**0.001
Type of toilet facility	Flush toilet	26.5	1,776	ref.		
	Pit toilet	33.5	4,482	1.09	(0.93–1.28)	0.285
	Hanging toilet	45.8	579	1.21	(0.95–1.54)	0.130
	No facilities (field, bush)	45.5	391	1.27	(0.97–1.68)	0.086
Salt iodization test outcome	0 PPM (not iodized)	38.1	1,907	ref.		
	More than 0 PPM and less than 15 PPM	38.2	1,443	1.12	(0.96–1.31)	0.146
	15 PPM or more	29.3	3,750	0.95	(0.82–1.09)	0.427
Number of under five children			7,230	0.99	(0.89–1.10)	0.847

Table 2 also shows the socioeconomic risk factors associated with the risk of child stunting, which we estimated using binary logistic regression. Estimated results suggest that increase in age is associated with the increased risk of stunting. Children aged 12 to <24 months had over twofold risk of stunting compared with children who were below 6 months [OR = 2.54; 95% CI: 2.21–2.92]. Gender of children was determined to be a significant predictor of stunting. Female children had 22% lower odds of stunting than their male counterparts [OR = 0.78; 95% CI: 0.70–0.87]. Geographical location, expressed in terms of geographical division, turned out to be another significant determinant of stunting. Our study found that children living in Sylhet division were 1.26 times more likely to be stunted than the children living in Dhaka division [OR = 1.26; 95% CI: 1.02–1.55]. Children from Khulna division [OR = 0.69; 95% CI: 0.57–0.84] and children from Rajshahi division [OR = 0.78; 95% CI: 0.64–0.96] had, respectively, 31% and 22% less likelihood of being stunted relative to children from Dhaka division. Lower levels of maternal education emerged as statistically a significant determinant of stunting, with lower maternal education being associated with a higher chance of child stunting. Mothers who did not complete secondary education had more likelihood of having stunted children than did mothers who had completed secondary education [OR = 1.47; 95% CI: 1.16–1.86]. Children of mothers who had completed primary education [OR = 1.68; 95% CI: 1.28–2.20] and children of mothers who had not completed primary education [OR = 1.84; 95% CI: 1.39–2.43] were both significantly more likely to be stunted than the children of mothers who had completed secondary education or higher. The risk of stunting among children whose mothers' had no formal education was almost two times higher compared with children of those mothers who had completed secondary education [OR = 1.96; 95% CI: 1.49–2.59]. Paternal education was another crucial determinant of stunting. Primary completed fathers had 31% more risk of having stunted children compared with children of fathers who had completed secondary education [OR = 1.31; 95% CI: 1.03–1.66]. Household position in wealth index was found to be another important predictor of child stunting. Our study estimated that children from the second quintile in wealth index had a 52% higher likelihood of stunting than the children from richest quintile [OR = 1.52; 95% CI: 1.18–1.97]. In comparison with children from the richest households, children from the poorest households had an approximately two times higher risk of develop stunting [OR = 1.96; 95% CI: 1.50–2.56]. Our study failed to determine any significant association of toilet facility and iodized salt with the risk of stunting. The study also found no significant relationship between the risk of stunting and either household size or number of under-five children in household.

### 3.3 Determinants of wasting

Table 3 exhibits the distribution of wasted children over socioeconomic and background characteristics of the study population. 12.5% children in households with a household member less than four years of age were wasted. Among three age groups, the highest proportion of wasted children was found in age group 12 to <24 months (12.9%). The proportion of male children wasted (13.1%) was substantially higher than the proportion of female children who were wasted (9.5%). The highest proportion of wasted children was found in Sylhet division (14.3%) followed by Barisal division (12.4%) and Chittagong division (11.9). Wasting prevalence was highest among children of uneducated mothers (13.9%) and the lowest percentage of wasting prevalence was found among the children of mothers who had completed secondary education (7.3%). Uneducated fathers also had the highest proportion of wasting prevalence among their children (12.9%). Only 6.7% children from the richest households were wasted, while a large proportion of children from the poorest households were wasted (14.2%). The percentage of wasted children from households with no toilet facility was estimated as 17.14%.

**Table 3:** Risk factors for wasting (WHZ scores < -2SD) among children aged less than twenty four months.

Factors		Wasted (%)	N	OR	95% CI	P value
Household member	<4	12.5	886	ref.		
	4 to <7	11.3	4,475	0.86	(0.68–1.09)	0.210
	7 or more	10.8	1,869	0.84	(0.64–1.12)	0.241
Age (Months)	0 to <6	9.4	1,758	ref.		
	6 to <12	10.2	1,813	1.10	(0.87–1.38)	0.446
	12 to <24	12.9	3,659	1.44	(1.18–1.76)	**<**0.001
Gender	Male	13.1	3,665	ref.		
	Female	9.5	3,565	0.68	(0.58–0.79)	**<**0.001
Area	Urban	10.4	1,153	ref.		
	Rural	11.5	6,077	0.91	(0.72–1.15)	0.426
Division	Dhaka	11.2	1,850	ref.		
	Barisal	12.4	677	1.03	(0.77–1.38)	0.849
	Chittagong	11.9	1,451	0.98	(0.78–1.24)	0.888
	Khulna	10.7	995	0.97	(0.75–1.26)	0.825
	Rajshahi	8.9	677	0.68	(0.50–0.94)	0.019
	Rangpur	10.4	916	0.76	(0.57–1.00)	0.051
	Sylhet	14.3	664	1.25	(0.93–1.67)	0.138
Education of mother	Secondary complete or higher	7.3	881	ref.		
	Secondary incomplete	10.4	2,705	1.37	(0.96–1.96)	0.081
	Primary complete	11.9	1,141	1.37	(0.91–2.04)	0.128
	Primary incomplete	13.0	996	1.37	(0.91–2.08)	0.132
	None	13.9	1,507	1.46	(0.97–2.19)	0.073
Education of father	Secondary complete or higher	8.5	1,057	ref.		
	Secondary incomplete	9.2	1,426	0.83	(0.61–1.14)	0.243
	Primary complete	12.1	978	1.01	(0.72–1.42)	0.943
	Primary incomplete	14.3	1,060	1.14	(0.81–1.59)	0.458
	None	12.9	2,145	0.93	0.67–1.29)	0.669
Wealth index quintile	Richest	6.7	1,056	ref.		
	Fourth	10.0	1,175	1.54	(1.07–2.21)	0.020
	Middle	10.8	1,364	1.59	(1.09–2.32)	0.017
	Second	12.2	1,571	1.74	(1.18–2.58)	0.005
	Poorest	14.2	2,064	1.79	(1.19–2.68)	0.005
Type of toilet facility	Flush toilet	9.1	1,776	ref.		
	Pit toilet	11.2	4,482	1.03	(0.81–1.30)	0.822
	Hanging toilet	15.0	579	1.17	(0.84–1.64)	0.361
	No facilities (field, bush)	17.1	391	1.58	(1.08–2.30)	0.017
Salt iodization test outcome	0 PPM (not iodized)	13.4	1,907	ref.		
	More than 0 PPM and less than 15 PPM	11.6	1,443	0.81	(0.65–1.01)	0.060
	15 PPM or more	10.3	3,750	0.84	(0.69–1.02)	0.080
Number of under five children			7,230	1.08	(0.92–1.25)	0.348

Table 3 also demonstrates the risk factors associated with child wasting, estimated using binary logistic regression model. According to our findings, children of age 12 to <24 months had significantly higher likelihood of wasting than the children aged less than 6 months [OR = 1.18; 95% CI: 1.18–1.76]. There were 32% lower likelihood of wasting among female children compared with male children [OR = 0.68; 95% CI: 0.58–0.79]. Our study revealed that children living in Rajshahi division were less likely to be wasted than the children living in Dhaka division [OR = 0.68; 95% CI: 0.50–0.94]. Household position in wealth index quintile appeared as significant predictor child wasting. It is evident from Table 3 that as we move along the wealth index quintile, the odds ratio increases, suggesting household position in lower wealth index is associated with higher risk of wasting among children below 24 months. Children from households which belonged to poorest category in wealth index had 79% larger likelihood of wasting compared with children from richest households [OR = 1.79; 95% CI: 1.19–2.68]. Children living in households which didn't have any toilet facility were more likely to be wasted than the children from households which had flush latrine as toilet facility [OR = 1.58; 95% CI: 1.08–2.30].

## 4 Discussion

In the present study, malnutrition among children below 2 years of age was assessed by calculating Z scores for two indicators: height for age (HAZ), and weight for height (WHZ) – widely used anthropometric measurement techniques of assessing malnutrition. Stunting in the first two years of life is critical, the duration between pregnancies to 24 months is vital for nutritional intervention to reduce the adverse effect on child survival, health and development (Victoria et al., 2008). In Bangladesh, the prevalence of stunting among 2-year old children declined from 44 percent in the year 1990 to 36.1 percent in the year 2014 (United Nations Children's Fund (UNICEF), 2016; BDHS, 2014) with an average annual rate of reduction of 4.5 percentage point. Regardless this remarkable achievement, 16% severely stunted children remains a strong challenge for Bangladesh (Ahmed et al., 2012). This study reports on the chronic malnutrition (stunting and wasting) and identifies the most significant socioeconomic, demographic, health and nutrition care factors of stunting and linear growth of children below 2 years of age in Bangladesh. Since this study included a large sample size (n = 7230 and covered a wide geographic area, it may reasonably be considered to reflect the condition of all children in Bangladesh under two years of age. Hence on the basis of the findings, policy and investment priorities can be determined.

In our study, stunting and wasting were predominant in the children of older age group. This study found that stunting rate of children of 12 to <24 months were almost two-fold higher than the infants of <6 months old Bangladesh. The height for age Z score (HAZ) progressively worsened with age indicating that nutritional impoverishment from infancy to early childhood increases rapidly after 12 months of age. Similarly, the prevalence of wasting was higher among children 12 to <24 months old than that of children 0 to <6 months old (9.4% and 12.9%, respectively). This can be explained by the fact that children of Bangladesh are exclusively breastfeed for five months (Haider et al., 2000), hence the sign of malnutrition express later when inadequate weaning practices compromise child's nutritional condition. This result was supported by other studies that demonstrated that stunting and poor linear growth progressively increased with age (Marriott et al., 2011; Frempong and Annim, 2017). A significant gender differential for linear growth and wasting was evident in this study. Although preference for male child is stronger in South Asian countries, our study estimated higher stunting and wasting rate among boys compared to girls. According to our study findings, 35.8% boys below 2 years were stunted where as the number was 31% for girls. Prevalence of wasting followed the similar path of higher wasting rate among boys compared to girls which is consistent with the previous studies from Pakistan, Ghana, and sub Saharan Africa (Hazarika, 2010; Hong, 2007; Wamani et al., 2007; Abera et al., 2019). The possible reason behind girls' lower stunting and wasting rate than boys can be explained by some factors. Firstly, discrimination against girls in the allocation of food and healthcare within the household is now not very common as it was earlier (Choudhury et al., 2000; Marcoux, 2002). Secondly, with gender differences, girls cope better than boys with less than sufficient amount of food (Marcoux, 2002).

Linear growth and stunting were less likely among children living in urban areas (30.2% vs. 34% in rural areas) and children from richest wealth index quintile (20.2% vs. 40.6% in the poorest wealth quintile). Economic inequality was found to be strongly associated with child chronic malnutrition since children in the poorest 60% households were at more than two-fold risk of being stunted than children in the richest 20% of households, independent of the characteristics of the children, mothers, household and other factors. This result confirmed that children of rural areas and poor are more likely to experience malnutrition. Living in poorer condition with inadequate food intake, lack of fundamental health services and greater risk to infections made the rural children prone to wasting and stunting than children of urban and better-off households. In addition to this the quality of antenatal care relates closely to the wealth. For example, according to the Bangladesh and Demographic Health Survey 2017 (BDHS) only 7% of pregnant women from the lowest wealth quintile received quality ANC compared with 37% of women from the highest wealth quintile (National Institute of Population Research and Training (NIPORT) and ICF, 2019) This result supported the findings from earlier research in other developing countries (Hong, 2007; Meshram et al., 2012; Hong and Mishra, 2016; Kien et al., 2016) and further confirmed that household economic condition is a key component of nutritional status of children under two years in developing countries.

In our analysis, parental education level has been identified as a significant factor in promoting nutrition of children under 2 years old. According to the findings of our study, mothers who had completed secondary or higher level of education had significantly lower stunted child than illiterate mother. An analogous result was found for paternal education. The result was supported by other studies which showed an association to parental education with improved growth outcomes of children in India, Thailand, Peru, Ghana, and Brazil (Meshram et al., 2012; Ruel and Menon, 2002; Armar-Klemesu et al., 2000; Monteiro et al., 2009). Parental education influences healthcare behaviours such as childhood vaccinations, family planning, visiting the local health clinics and vitamin A supplementation (Semba et al., 2008). Besides, there is a general consensus that higher education leads to higher income. Hence, increasing household earnings enables parents to invest more on health care services and proper food intake for their children (Chowdhury et al., 2018) might be the reason of lower stunting rate of educated parents.

Our analysis by geographical location, expressed in division (administrative regions), demonstrates that geographical location is a significant predictor of stunting and poor linear growth. Both stunting and wasting were highest in Sylhet division among the seven divisions in Bangladesh (40.1% and 14.3% respectively). The odds ratio to be stunted among children of Chittagong division, Rangpur division, and Sylhet division were respectively 1.06, 1.03 and 1.26 times more likely compared to the children of Dhaka division. Children from Khulna division and Barisal division had, respectively 31% and 23 % fewer likelihood of being stunted relative to that of Dhaka division. Although Khulna and Barisal divisions are not economically advanced compared with other divisions, better nutritional status in these regions can be explained as the result of Social Safety Net Programs launched in those areas. Khulna and Barisal divisions are located to the south of the country and being coastal area, they are the worst sufferer of climate change. To reduce the climate risk and nutritional deficiency due to frequent natural disasters, these two geographical regions are given highest priority for governmental and non-governmental aid. Data on Social Safety Net Programs (SSNP) shows that the highest proportion of households (37.3 %) received benefits from SSNP in Khulna division followed by 34.4 % households from Barisal division (Barkat et al., 2013). It is therefore recommended that geographical targeting in the means of Social Safety Net Program might possibly improve nutritional conditions in other regions. Socio-economic status, cultural values, social security, poor accessibility to education and health services for mother and child may be the causes of regional differences in stunting and wasting of children.

## 5 Conclusion

The causes and influencing factors of stunting and wasting among children less than two years is manifold and complex in nature. However, this study found evidence that socioeconomic and demographic variables have significant influence on stunting and wasting of children below 24 months. According to the findings of the study, policy makers might focus on issues particularly, parental education, improved sanitation facilities, households' economic wellbeing especially on nutrition and food security, and awareness and promotion of breast-feeding and complementary feeding of young children (6–24 months). Moreover, poorer households, families having male children and households prioritized by geographical location can be targeted for policy intervention for a better outcome.

The study also found a strong association between stunting and wasting of children within the complementary feeding period among the sample. It is particularly important to develop a better understanding of infant and young child feeding practices and how these relate to children's growth and development in the first 2 years of life. Additionally, considering the quality of complementary food, disseminating knowledge and formulating effective programs to encourage mothers to continue breast feeding as a supplementary after 6 months of age are some other measures could be taken in this regard. The findings of the study urge the importance of parental education. Strategies might be developed to increase parents' education and awareness to improve nutrition and reduce both stunting and wasting which is evident from the study. The current paper also suggests measures to emphasize on hygienic latrine, improve socioeconomic status of households and intervention sorted by geographical location where stunting and wasting is prevalent to reduce malnutrition.

## Declarations

### Author contribution statement

Tuhinur Rahman Chowdhury, Muntaha Rakib, Sabiha Afrin, Sue Saltmarsh, Stephen Winn: Analyzed and interpreted the data; Wrote the paper.

Sayan Chakrabarty: Conceived and designed the experiments; Analyzed and interpreted the data; Wrote the paper.

### Funding statement

This research did not receive any specific funding from agencies in the public, commercial, or not-for-profit sectors.

### Competing interest statement

The authors declare no conflict of interest.

### Additional information

Data associated with this study has been deposited at http://microdata.worldbank.org/index.php/catalog/2533.
